# Anti-inflammatory monocytes—interplay of innate and adaptive immunity

**DOI:** 10.1186/s40348-018-0083-4

**Published:** 2018-04-03

**Authors:** Georg Varga, Dirk Foell

**Affiliations:** Department of Pediatric Rheumatology and Immunology, University Hospital Muenster, University of Muenster, Domagkstr. 3, 48149 Münster, Germany

## Abstract

Monocytes are central to our health as they contribute to both hemispheres of our immune system, the innate and the adaptive arm. Sensing signals from the outside world, monocytes govern the innate immunity by initiating inflammation, e.g., through production of IL-1β. Uncontrolled and sustained inflammation, however, leads to auto-inflammatory syndromes and sometimes to autoimmune diseases. Monocytes can be a driving force for the establishment of such diseases when their ability to also contribute to the resolution of inflammation is impaired. It is therefore of vast importance to gain knowledge about the anti-inflammatory mechanisms monocytes can use to participate in downregulation and resolution of inflammation. Here, we summarize some of the known anti-inflammatory mechanisms and features of regulatory monocytes and shed light on their importance in governing innate and adaptive immune responses. Considering anti-inflammatory mechanisms of monocytes will also help to develop new strategies to use monocytes as therapeutic targets in the future.

## Introduction

Our immune system is perpetually searching danger signals to recognize and combat invading pathogens. Blood monocytes are among the first line of host defense and are equipped with pattern recognition receptors (PRRs), such as toll-like receptors (TLRs) and NOD-like receptors (NLRs), to detect and respond to infection-associated pathogen-associated molecular patterns [1–3]. After inflammatory response is triggered, adaptive immunity becomes activated to effectively protect the host and to establish a memory immune response. Although inflammation is required for clearance of infection, excessive and chronic inflammation are energetically expensive and, uncontrolled, harm the host. Therefore, timely and efficient resolution of inflammation is essential. The resolution of immune response leading to restoration of homeostasis is an active regulatory process that is initiated already by inflammation itself [2, 4]. Besides neutrophils and macrophages that are considered the major cell types during the resolution phase, recently, it has become apparent that so-called regulatory monocytes can develop to suppress inflammatory responses and even adaptive immune cells [5]. The term regulatory monocytes is not precisely defined yet, but among the first monocytic cells described to be suppressive to T cells were the so-called monocytic myeloid-derived suppressor cells (mo-MDSCs). Discovered to develop in tumor environments, MDSCs become more and more apparent to play more general roles in immune suppression and regulation (reviewed in [6]). However, due to their high plasticity, peripheral monocytes in general are comprehensively suited to either develop to inflammation triggering cells or to mediate a shift to resolution. Mainly pro-inflammatory at the beginning of infection, blood monocytes can subsequently be reprogrammed from an activated to a suppressive phenotype. Here, monocytes show a shifted rather than a shutdown gene expression and undergo a transition from inflammation to tolerance [7, 8]. In this review, we will summarize our current knowledge about regulatory monocytes, other than MDSC, and mechanisms that allow the conversion of potentially inflammatory monocytes into their regulatory companions.

### IL-1β-induced reprogramming of monocytes

**Fig. 1:**
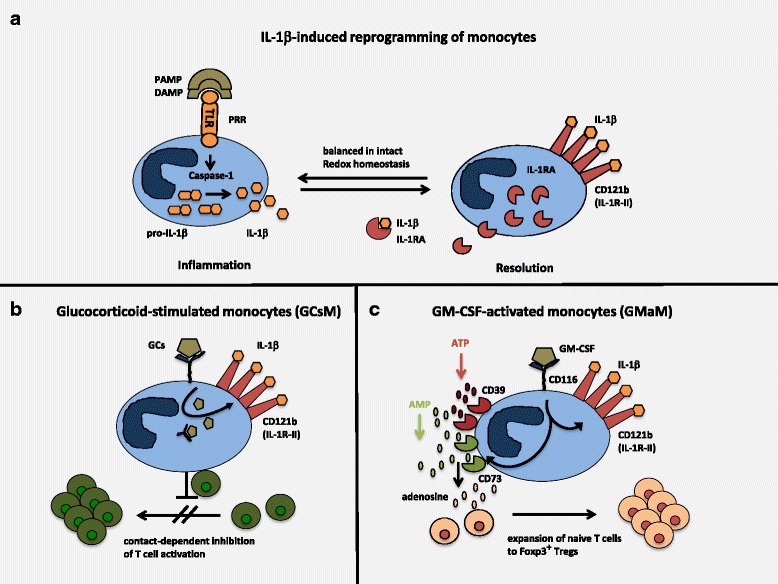
Reprogramming of monocytes into anti-inflammatory cells to regulate inflammation and adaptive immunity. **a** IL-1 β-induced reprogramming of monocytes: upon encounter with PAMPs (pattern-associated molecular patterns) or DAMPs (danger-associated molecular patterns), so-called PRRs (pattern recognition receptors), here TLR (toll-like receptor) transmits signals into the cell that activate caspase-1. Active caspase-1 cleaves immature pro-IL-1 β into mature IL-1 β which then is released into the environment to induce inflammation. In balanced redox homestasis, prolonged activation of monocytes leads to more increased production of counterbalancing IL-1RA (IL-1 receptor antagonist) and upregulation of IL-1 decoy receptor CD121b (IL-1R-type II). Both bind and neutralize pro-inflammatory IL-1 β and contribute to the resolution phase of the inflammatory response. **b** Glucocorticoid-stimulated monocytes (GCsM): GCs (glucocorticoids) are sensed by GC receptors and induce reprogramming of monocytes to anti-inflammatory cells. Upregulation of IL-1 decoy receptor CD121b is one hallmark of GCsM. In contact with adaptive immunity, here T cells, GCsM efficiently downregulate T cell activation through yet unidentified mechanisms. In vivo, in a model of T cell-induced colitis, this leads to complete remission of inflammatory tissue damage [13]. **c** GM-CSF-activated monocytes (GMaM): GM-CSF is bound by the GM-CSF receptor CD116 on monocytes. Likewise, also GM-CSF reprograms monocytes into anti-inflammatory cells but with different features to confer regulation of inflammatory response. While upregulation of CD121 is similar to GCsM, increased expression of the exo-enzymes CD39 and CD73 is unique to GMaM. Using active CD39 and CD73 GMaM are capable to breakdown extracellular ATP into AMP and subsequently to adensoine that is known to help differentiation of Tregs. In contact with naive T cells, this allows GMaM to induce the differentiation and expansion of of Foxp3^+^ Tregs. Therapeutical use of GMaM in a mouse model of colitis showed complete suppression of inflammation in vivo, presumably through induced Tregs [15, 16]

A strong pro-inflammatory cytokine like IL-1 β not only initiates pro-inflammatory response but can also induce a switch to a subsequent tolerogenic phase. In a study with PBMCs from healthy human donors, Giesbrecht et al. could demonstrate that monocytes that had encountered TLR7/8 stimulation in a first phase could be reprogrammed to regulatory cells under the influence of IL-1 β and IL-6. Thus, depending on the phase of activation, IL-1 β and IL-6 could be considered as mediators of resolution of inflammation that is mediated through monocytes [9]. This can be especially important during sterile inflammation where IL-1 β and IL-1RA (IL-1 receptor antagonist) play decisive roles in driving or counterbalancing autoinflammation. In addition, the IL-1 receptor type II (CD121b) can be upregulated to function as a decoy receptor for IL-1 β. In case of redox distress and subsequent imbalance of these IL-1 components, those monocytes can be a driving force to autoinflammation. Reducing redox distress here leads to increased production of IL-1 RA by monocytes and consequently results in lower inflammation (illustrated in Fig. 1a) [10].

### Glucocorticoids induce anti-inflammatory monocytes

Using gene array technology, we could establish that glucocorticoids, that are among the most widely used anti-inflammatory drugs, would reprogram monocytes to confer anti-inflammatory functions in human and murine monocytes [11–13]. Phenotypically, with upregulated expression of IL-4R alpha chain CD124, glucocorticoid-stimulated monocytes (GCsM) resemble MDSCs [11], but it needs to be shown whether they are in terms of mode of action. One hallmark of GCsM is the upregulation of IL-1 receptor II (CD121), and this has not been described for MDSCs so far. Even more, in a mouse model of inflammatory bowel disease (IBD), GCsM were able to heal established colitis by downregulating activated T cells (see Fig. 1b) and by inducing the accumulation of Tregs at sites of inflammation in the colon [13]. Thus, GCsM might become ex vivo targets for GC-induced therapies in order to avoid systemic effects of glucocorticoids (GCs) in vivo by substituting with GCsM.

### Reprogramming of monocytes by GM-CSF to induce regulatory monocytes

We recently demonstrated activation-dependent cell death in human monocytes, when additional pro-inflammatory signals (e.g., IFNγ) synergize with GM-CSF to over activate monocytes. Whether this is a mechanism to self-limit accelerated inflammation is currently unknown [14]. However, when monocytes are stimulated with GM-CSF alone, they become reprogrammed to anti-inflammatory monocytes that are capable to downregulate intestinal inflammation in a mouse model in vivo via induction of Tregs [15]. Other than with the GCsM, GM-CSF-activated monocytes (GMaM) induced Foxp3^+^ Tregs de novo from naive T cells utilizing extracellular ATP for degradation into adenosine. Exo-enzymes CD39 and CD73 are upregulated and used by GMaM to generate adenosine that directly differentiates naive T cells into Tregs. Furthermore, also GMaM upregulate the IL-1 decoy receptor CD121b [16]. Thus, monocytes can be therapeutical targets to be reprogrammed into anti-inflammatory cells (summarized in Fig. 1c) in vitro and in vivo.

## Conclusions

Research recently has revealed not only that monocytes are part of inflammation and inflammatory disease, but also can be reprogrammed to the opposite, namely to cells that are highly anti-inflammatory and contribute to resolution of inflammation. Knowing the mechanisms how monocytes can be turned into anti-inflammatory cells will allow for targeted therapeutic intervention using these pathways to down-modulate inflammation in vivo.
